# Reward Processing under Chronic Pain from the Perspective of “Liking” and “Wanting”: A Narrative Review

**DOI:** 10.1155/2019/6760121

**Published:** 2019-04-21

**Authors:** Xinhe Liu, Ning Wang, Lijia Gu, Jianyou Guo, Jinyan Wang, Fei Luo

**Affiliations:** ^1^CAS Key Laboratory of Mental Health, Institute of Psychology, Chinese Academy of Sciences, Beijing 100101, China; ^2^Department of Psychology, University of Chinese Academy of Sciences, Beijing 100049, China

## Abstract

The therapeutic goals of patients with chronic pain are not only to relieve pain but also to improve the quality of life. Chronic pain negatively affects various aspects of daily life, such as by decreasing the motivation to work and reward sensitivity, which may lead to difficulties in daily life or even unemployment. Human and animal studies have shown that chronic pain damages reward processing; the exploration of associated internal mechanisms may aid the development of treatments to repair this damage. Incentive salience theory, used widely to describe reward processing, divides this processing into “liking” (reward-induced hedonic sensory impact) and “wanting” (reward-induced motivation) components. It has been employed to explain pathological changes in reward processing induced by psychiatric disorders. In this review, we summarize the findings of studies of reward processing under chronic pain and examine the effects of chronic pain on “liking” and “wanting.” Evidence indicates that chronic pain compromises the “wanting” component of reward processing; we also discuss the neural mechanisms that may mediate this effect. We hope that this review aids the development of therapies to improve the quality of life of patients with chronic pain.

## 1. Introduction

Chronic pain is a worldwide problem for which effective treatment is lacking [1]. It is associated with physical disability, cognitive impairment, and negative psychological states, including anxiety, depression, and an increased risk of suicide [2–4]. Thus, chronic pain is composed of long-term physical and psychological pain, and an important goal of its treatment is to improve patients' quality of life [5]. Chronic pain-induced psychological changes may interfere with the processing of rewards, defined as “objects or events that generate approach and consummatory behavior, produce learning of such behavior, represent positive outcomes of economic decisions and engage positive emotions and hedonic feelings” (page 1, second paragraph) [6]. Given this definition, reward processing has several components, including the noticing of reward-related cues, bursts of motivation, hedonic perception, and reinforcement learning [7, 8]. Patients with chronic pain may lose pleasure in life and/or motivation to work [9, 10], decreasing their sensitivity to rewards [11], but the mechanism by which chronic pain affects reward processing remains unclear. Exploring changes in reward processing under chronic pain may facilitate the improvement of treatment.

Researchers have used the incentive salience theory to explain reward processing during depression. This approach divides reward processing into “liking” and “wanting,” both of which are decreased under major depression [12–14]. “Liking” is the pleasurable experience derived from sensory input, usually dependent on reward stimulus' properties, and “wanting” is the internal motivational component related to the acquisition of reward stimuli [15]. In this review, we summarize studies of reward processing during chronic pain and discuss related neural mechanisms from the perspective of incentive salience theory.

## 2. Reward-Related Behaviors in Patients with Chronic Pain

### 2.1. Natural Reward-Related Behaviors during Chronic Pain

Studies conducted with healthy volunteers have shown that acute physical pain can increase the motivation to attain rewards under laboratory conditions [16, 17]. However, natural reward-related behaviors in patients with chronic pain may be more complicated. In a cross-sectional study, Geha and colleagues [11] found reduced hedonic perception of palatable food in patients with CLBP; “liking” ratings for palatable puddings were significantly lower among patients with CLBP than among controls, whereas sensory ratings of intensity, sweetness, creaminess, fattiness, and oiliness, as well as “liking” ratings for sugar-containing drinks, were similar in the two groups. These results indicate that patients with CLBP had normal senses of taste, but decreased food-related pleasure. In another study, patients with chronic back pain had lower detection thresholds for gustatory stimuli (i.e., bitter, salty, sweet, and sour) than did controls, with no significant difference in “pleasant” and “unpleasant” ratings for these stimuli between groups [18]. Thus, whether chronic pain affects the derivation of pleasure from gustatory stimuli remains a matter of debate.

### 2.2. Reward Sensitivity in Patients with Chronic Pain

The term “reward sensitivity” is used to describe positive beliefs about the probability of future rewards [19]. High reward sensitivity helps to identify and motivate repetition of pleasurable activities, whereas low reward sensitivity reduces the motivation to engage in such activities and facilitates the development or aggravation of depressive symptoms [20]. Anhedonia, a typical symptom of depression, is defined as loss of sensitivity to pleasure and motivation to acquire reward [13, 19]. In early studies, the 66-item Physical Anhedonia Scale (PAS) was used to assess physical anhedonia symptoms in patients with chronic facial and low back pain [21, 22]. PAS items encompass loss of the ability to feel pleasure from eating, drinking, touching, sex, temperature, smells, and sounds. Thus, the PAS inventory assesses mainly the capacity to hedonically perceive events and stimuli during daily activity. Patients with chronic pain reported high levels of physical anhedonia in these studies, suggesting that chronic pain reduces the emotional pleasure response.

The Behavioral Activation Scale/Behavioral Inhibition Scale (BAS/BIS), based on these behavioral approach system/behavioral inhibition system characteristics [23], has been used to assess reward sensitivity in human subjects. The behavioral approach system is sensitive to reward signals, and the behavioral inhibition system could be activated by aversive stimuli [19, 24]. Using the BAS/BIS scales, Elvemo et al. [25] found reduced reward responsiveness, defined as tendency to respond positive in the context of desired events or cues of potential future reward, in female patients with chronic idiopathic, visceral, and musculoskeletal pain, but no difference in reward drive, defined as the tendency to pursue reward, between these patients and a control group. They also observed significant higher physical anhedonia in chronic pain patients measured with the Beck Depression Inventory II. Becerra-García and Robles Jurado [24] used questionnaire, based on the behavioral approach system, to assess reward sensitivity in female patients with fibromyalgia, finding significantly reduced behavioral approach system activity, which could be interpreted as poor response to environmental incentives and reduced reward approach behavior. These studies have revealed the correlation between chronic pain and low reward sensitivity.

### 2.3. Goal Pursuit Behaviors in Patients with Chronic Pain

Researchers have examined motivation through assessment of the pursuit of goals [26–29], conceptualized as “internal representations of desired states, where states are broadly construed as outcomes, events, or processes” (Page 338, paragraph 1) [26]. Goal pursuit may be related to reward processing [30, 31], and its relationship to chronic pain has been explored using the daily diary method. Hardy et al. asked women with fibromyalgia to record feelings of pain, emotional distress, and fatigue and to rate goal conflicts in daily activity, including household and job tasks and interpersonal relationships, using daily diaries [32]. They found that goal pursuit behaviors resulted in greater subjective feelings of pain and that emotional distress mediated the perception of goal conflicts. Mun et al. used the daily dairy method to assess daily goal conflicts in patients with chronic pain [10], finding that pain intensity was related positively to interference with work goals and that this effect was moderated by pain acceptance. Semistructured interviews have also been used to investigate daily goal conflicts in patients with fibromyalgia, revealing more self-reported pain-induced goal conflicts (most commonly affecting household, social, and interpersonal goals) in these patients than in healthy individuals [28]. These studies show that chronic pain inhibits daily goal pursuit activities. Under chronic pain, some short- and long-term goals may become unattainable [27], which may lead to the loss of positive feedback and attenuate the reward processing of goal pursuit behaviors. However, the direct relationship between reward processing and goal pursuit behaviors during the chronic pain remains to be discussed.

The aforementioned studies are summarized in Table 1. These researchers adopted different experimental paradigms, and the results consistently indicated that chronic pain disrupted various aspects of reward processing. Under some conditions, the effects of chronic pain on the emotional and sensory aspects of reward processing may be separate [11, 18]. Moreover, chronic pain and its management were found to affect daily activities by inducing more goal conflict, which may lead to the adaptive reduction of reward-seeking motivation. In the following sections, we introduce incentive salience theory and use this perspective to present research on reward processing under chronic pain. As only few human studies have directly explored the relationship between chronic pain and reward processing using incentive salience theory, we describe here mainly evidence derived from animal research in the following chapters. Although animal studies are not entirely a representative of human studies, they may provide important reference for future human studies and facilitate the understanding of how chronic pain affects reward processing.

## 3. Reward Processing during Chronic Pain in Animal Studies

### 3.1. Incentive Salience Theory

Incentive salience theory has been applied widely to explain reward processing in individuals with psychiatric disorders, such as drug addiction, gambling disorders, overeating [15, 33, 34], major depression [12–14], and schizophrenia [35]. Within the framework of this theory, “wanting” can be motivated by reward-conditioned cues, and thus may involve associative learning, attention, and memory retrieval. As “wanting” can be translated into motivation and bursts of reward-seeking behavior (i.e., approach behaviors and attempts to obtain stimuli), some researchers refer to it as “incentive salience” [15, 36, 37]. In the context of the consumption of delicious food, “liking” is the pleasurable emotional experience induced via the activation of peripheral sensory (gustatory, tactile, and/or olfactory) receptors, which triggers nerve impulses and activates the reward circuit [38]; “wanting” is the internal desire and wish to repeat the experience triggered by the smell or sight of the food [15, 39].

“Liking” and “wanting” have been measured using specific experimental paradigms. As the hedonic experience dominates during the consumption of reward stimuli [40], the pleasurable sensory stimulus of sweetness has often been used to measure “liking.” The sucrose preference test (SPT) measures the consumption of sucrose-containing water, which could reflect the pleasurable sensory input of this activity in animal studies [13]. Taste reactivity is another classical paradigm used to measure “liking,” through the scoring of animals' orofacial expressions and behavior during the consumption of sucrose-containing water; tongue protrusion and paw licking, for example, are regarded as behavioral indices of pleasure [36, 41, 42]. A more diverse set of paradigms is used to measure “wanting,” as the degree of motivation to acquire reward stimuli. Experiments have involved reward-seeking or goal pursuit tasks [15, 43], such as T-maze navigation for rats [41]. The self-administration (SA) model with natural rewards or addictive drugs has also been used widely to measure “wanting” through quantification of the amount of reward earned in rats (e.g., by lever presses or nose pokes) [44–46]. “Wanting” has also been measured by quantifying rats' daily consumption of food or water [43, 47, 48].

### 3.2. Effects of Chronic Pain on “Liking”-Related Behaviors

Animal studies of the effect of chronic pain on reward-related behaviors are summarized in Table 2. As the decreased preference for sucrose in the SPT is often explained as anhedonia, this test has been used widely to examine the comorbidity of chronic pain and depressive emotion in animal research. Several studies have shown that chronic pain decreases rodents' preference for sucrose-containing water. For example, Dellarole et al. reported that chronic constriction injury (CCI) in mice induced neuropathic pain that decreased the 1% sucrose-containing water preference, reduced body weight, and degraded the physical state of the rats' coats; this process was combined with neuroplastic remodeling in the hippocampus, a key emotional area [49]. Thus, the authors concluded that CCI induced depressive emotion. Wang et al. reported that spared nerve injury (SNI) induced neuropathic pain that decreased rats' preference for sucrose-containing water [50]. Neuropathic pain has also been found to attenuate the hedonic perception of sweetness in mice [51, 52]. Reduced sucrose preference has also been observed in other chronic pain models, such as those of monoarthritis [64] and fibromyalgia [53].

Other animal studies employing the SPT have revealed no reduction of sucrose preference during chronic pain [60, 61]. Urban and colleagues investigated the daily behaviors of mice, including their hedonic perception during the SPT [57]. After SNI or complete Freund's adjuvant (CFA) injection, the animals' preference for water containing 2% sucrose, daily food and water consumption, and weight remained unchanged for weeks. Shi et al. used the SPT to explore the interaction between chronic pain and depression [54], but they observed that spinal nerve ligation (SNL) alone was not sufficient to decrease rats' preference for water containing 1% sucrose. In other researches, Shi et al. also reported CFA-induced chronic pain inflammatory is not enough to decrease the sucrose preference [55]. Bravo et al. examined depressive symptoms in rats with CCI and similarly found that neuropathic pain did not affect the preference for sweet cereals, food consumption, or body weight [58].

Thus, results regarding the effects of chronic pain on “liking” are inconsistent. One possible reason is that the SPT is not a stable index and can be influenced by many factors, such as experimental conditions, animal types, and chronic pain models. For example, some researchers performed the SPT within 2 weeks after the induction of chronic pain models [56, 59], which may be an insufficient time period to allow for alteration of the “liking” component.

### 3.3. Effects of Chronic Pain on “Wanting”-Related Behaviors

Chronic pain has shown divergent effects on “wanting” behavior, according to the experimental protocol used (Table 2). Some researchers have adopted SA models to explore the effects of chronic pain on reward-seeking behaviors using fixed ratio (FR) and progressive ratio (PR) protocols. Under the FR protocol, animals must press a lever or perform a nose poke a fixed number of times to earn a reward. Under the PR protocol, the number of actions required to obtain the reward increases, making reward acquisition progressively more difficult. Hipólito and colleagues reported that CFA injection-induced inflammatory pain did not affect rats' sucrose pellet SA behavior under an FR protocol, but that this behavior was attenuated under a PR protocol with sucrose pellets or 50 *µ*g/kg heroin, but not the seeking behaviors to high dose of heroin (200 *µ*g/kg) [62]. Schwartz et al. trained mice to nose poke to obtain reward pellets and built chronic pain models with CFA injection and SNI, respectively [61]. They observed that these treatments decreased the nose poke behavior to food pellets under a PR protocol, but did not affect nose poke behavior or daily food consumption under an FR protocol. Similarly, Okun et al. observed that CFA injection-induced inflammatory pain transiently decreased SA lever pressing in rats under a PR protocol, whereas SNL did not affect SA behavior or daily water or food consumption under an FR protocol [60].

Decreased reward-seeking motivation under chronic pain has also been demonstrated using the pain avoidance-reward approach task. Schwartz et al. [63] trained rats to seek small and large rewards (water containing 3% and 10% sucrose, respectively) with the probability of experiencing laser-induced heat pain. The rats maintained SA behavior for both sucrose concentrations while ignoring the heat pain attacks. After SNI, neuropathic pain reduced SA behavior for the small reward, but did not affect large reward seeking. These results might be interpreted as reflecting the motivational value of reward decrease under chronic pain.

Differences in the difficulty of animals' reward acquisition among experimental paradigms (i.e., between FR ad PR protocols) may have led to the divergent results regarding the effect of chronic pain on “wanting” behavior. Most SA studies performed with PR protocols have shown that chronic pain decreases “wanting,” whereas those performed with FR protocols have shown no effect on SA behavior or daily food or water intake [57, 58, 59], which may reflect the subjective perception of the effort required to complete the task. Researchers believe that, at a certain breakpoint (BP), animals stop nose poking or lever pressing because they perceive that earning the reward is no longer worth the effort. Thus, the BP has been considered to reflect the internal motivation to obtain the reward, which is measured more suitably with a PR protocol than with an FR protocol or the measurement of spontaneous food and water intake [65]. Therefore, we consider that SA models with PR protocols reflect more advanced “wanting” components, which are much more sensitive to chronic pain.

## 4. Modulatory Mechanisms of Chronic Pain on Reward Processing

Pharmacological activation of the dopamine (DA) system, which is one kind of the neurotransmitter closely related to reward, by amphetamine has been found to enhance food intake (“wanting”), but to have no effect on taste reactivity (“liking”) in rats [41, 48, 66, 67]. The role of the opioidergic system in reward processing has also been examined; pharmacological stimulation of mu, delta, and kappa receptors in the anterior-posterior nucleus accumben (NAc, a hedonic “hotspot”) increased tasty reactivity scores and food intake [43, 66, 68].

Many studies have shown that chronic pain can alter the DA and opioidergic systems. In rats, chronic inflammatory and neuropathic pain reduced morphine-induced conditioned place preference (CPP), increased the expression of kappa receptors in the NAc, and downregulated mu receptors in the ventral tegmental area (VTA). These processes were combined with suppression of morphine-induced DA release in the NAc [69–71]. Hipólito et al. reported that CFA-induced inflammatory pain downregulated the expression of mu receptors in the VTA and reduced the baseline DA level in the NAc, which had the combined effect of reducing food SA behavior [62]. Human research also showed the mu receptors in patients with chronic pain were downregulated [72, 73].

Otherwise, findings from a recent animal study suggest that D2-like receptors are involved in reward-seeking behavior. Specific ablation of medium spiny neurons that express D2-like receptors (D2-MSNs) in the ventrolateral striatum reduced goal-directed behavior in mice, executing a three-choice serial reaction-time task [74]. Optogenetic inhibition of D2-MSNs before lever presentation in an SA model also decreased the BP under a PR protocol in rats [74]. Another recent study showed that the downregulation of D2-MSNs under chronic pain correlated with decreased reward-seeking motivation in mice; SNI and CFA injection inhibited the excitatory postsynaptic potential of D2-MSNs, but not that of D1-MSNs, in the NAc [61]. The authors also found that the food-seeking behavior of these animals was inhibited under a PR protocol, but not under an FR protocol; sucrose-containing water preference and daily food consumption were unchanged. Neuroimage results from human research also support that D2-like receptor was downregulated by chronic pain. A PET study showed decreased availability (dysfunction) of D2-like (D2 and D3) receptors in the ventral striatum in patients with chronic nonneuropathic back pain [75]. Similar observations were made in female patients with fibromyalgia [76].

“Wanting”- and “liking”-related brain structures have been reviewed by Robinson et al. [15]. They proposed that the “liking” and “wanting” brain systems overlapped in mesolimbic structures. Pharmacological disruption of NAc could decrease the “wanting” behaviors such as spontaneous food and water intake [41, 48, 66, 67]. Besides, Schwartz et al. have reported that the neuropathic pain could decrease the neural activities in the mPFC-NAc pathway and suppress the reward-seeking behaviors in the pain avoidance-reward approach task in rats [63]. The above studies indicate the potential neural mechanism that mediate the inhibition of reward processing in chronic pain, including DA system, mu receptor, D2-like receptor, and mPFC-NAc pathway; however, further experiments are still needed to explore these mechanisms.

## 5. Limitations

The main limitation of this study is related to the differences between animal and human studies of changes in reward-related behaviors and neural mechanisms. For example, Pool et al. argue that differences in the definitions of “wanting” and “liking,” which are more conflicted in humans than in animal research, lead to the use of animal-based experimental designs that do not distinguish “wanting” and “liking” in humans [40]. Experimental results from animal research can provide reference, but could not equal with results from human research and could not apply the conclusion to human research. Systematic human research needs to be performed to acquire more evidence on reward processing in the context of chronic pain.

## 6. Conclusion and Future Directions

In this review, we employed incentive salience theory to describe evidence on reward processing under chronic pain. The distinction of “liking” and “wanting” components of reward processing may aid discrimination of the effects of chronic pain on pleasurable sensory input and reward-seeking motivation. Based on evidence accumulated to date, we conclude that chronic pain reduces “wanting” behaviors. Bursts of reward-seeking behavior involve cognitive processes such as attention, working memory, and associative memory retrieval. Patients with chronic pain are sensitized to pain-related information, and pain management occupies their attentional resources [77, 78]. As a result, long-term chronic pain-induced attentional impairment might conflict with reward-seeking behavior, leading to the adaptive reduction of “wanting.” In addition, some studies have shown that chronic pain can decrease “liking” behaviors.

The effect of chronic pain on reward processing seems to differ between the sexes. Chronic pain has been found to decrease reward-related behaviors (i.e., sexual behavior and sucrose preference) in female, but not in male, animals [79, 80]. Sexual hormones may mediate reward processing, given the higher prevalence of food addiction and drug abuse in female than in male rats [81]. In human research, females are also more vulnerable to chronic pain than males [82, 83]. Future studies should thus explore sex differences in the vulnerability of reward processing to chronic pain.

The “liking” and “wanting” perspective may provide a new way of thinking about reward processing under chronic pain. First, according to this perspective, reward sensitivity and reward-seeking motivation can change without affecting each other. Therefore, this framework could explain fundamental research findings that chronic pain does not decrease the ability to discriminate pleasurable sensory input, but does reduce reward-seeking behavior [60, 61]. Second, this perspective can be used to examine the comorbidity of chronic pain and drug addiction. Incentive salience theory has been used widely to explain drug addiction in humans and animals; for example, some addictive drugs sensitize the “wanting” system, resulting in compulsive drug-seeking behavior [15, 81]. Patients with chronic pain are more likely to have contact with analgesics such as morphine and heroin, increasing the risk of drug addiction. Third, this perspective may enhance targeted drug treatment to improve reward processing in patients with chronic pain, as beyond psychological dissociation, “wanting” and “liking” are distinct in terms of brain structure and neurochemistry. Finally, as chronic pain is a worldwide problem that severely compromises the quality of daily life, further research should be conducted to explore whether this perspective can be applied to improve the well-being of patients with chronic pain.

## Figures and Tables

**Table 1:** Reward-related behaviors in patients with chronic pain.

Study	Disorders	Test	Measurement	Results
Geha et al. [11]	Back pain	Rating sugary drinks	Liking	No change
			Wanting	No change
			Sweetness	No change
			Intensity	No change
		Rating fat puddings	Liking	Decrease
			Wanting	No change
			Sweetness	No change
			Intensity	No change
Small and Apkarian [18]	Low back pain	Rating sucrose solution	Pleasantness	No change
			Intensity	Increase
Marbach and Lund [21]	Facial pain and TMJ pain	PAS	Anhedonia	Increase
Marbach et al. [22]	Back pain or facial pain	PAS	Anhedonia	Uncorrelated to pain intensity
Elvemo et al. [25]	Various	BIS/BAS	Reward drive	No change
		BDI	Anhedonia	Increase
		BIS/BAS	RER	Decrease
Becerra-Garcia and Robles Jurado [24]	Fibromyalgia	SPSRQ	RER	Decrease
Claes et al. [28]	Fibromyalgia	Semistructured interview	Goal conflicts	Increase
Hardy et al. [32]	Fibromyalgia	Daily diary	Goal conflicts	Increase
Mun et al. [10]	Not mentioned	Daily diary	Goal conflicts	Increase

Abbreviations: TMJ, temporomandibular joint; PAS, Physical Anhedonia Scale; BIS, Behavioral Inhibition Scale; BAS, Behavioral Activation Scale; BDI, Beck Depression Inventory; SPSRQ, Sensitivity to Punishment and Sensitivity to Reward Questionnaire; RER, reward-induced emotional responsiveness.

**Table 2:** Reward-related behaviors in animals with chronic pain.

Study	Species	Reward stimuli	Pain model	Test (results)
Dellarole et al. [49]	Mouse	SW	CCI	SPT (decrease)
Wang et al. [50]	Rat	SW	SNI	SPT (decrease)
Wu et al. [51]	Mouse	SW	SNI	SPT (decrease)
Bura et al., 2013	Mouse	SW	PSNL	SPT (decrease)
Amorim et al. [52]	Rat	SW	Arthritis	SPT (decrease)
Liu et al. [53]	Rat	SW	Fibromyalgia	SPT (decrease)
Shi et al. [54]	Rat	SW	SNL	SPT (no change)
Shi et al. [55]	Rat	SW	CFA	SPT (no change)
Su et al. [56]	Rat	SW	SNI; CFA	SPT (decrease)
Urban et al. [57]	Mouse	SW; food; water	SNI; CCI; CFA	SPT (no change); food intake (no change); water intake (no change)
Bravo et al. [58]	Rat	Cereals	CCI	Food intake (no change)
Goffer et al. [59]	Rat	SW; water	SNI	SPT (decrease); water intake (no change)
Okun et al. [60]	Rat	Food	CFA, SNL	SA with PR (CFA decrease, SNL no change); SA with FR (no change); tasty reactivity (no change)
Schwartz et al. [61]	Mouse	SW; food	CFA; SNI	SPT (no change); SA with PR (decrease); SA with FR (no change); food intake (no change)
Hipólito et al. [62]	Rat	SW	CFA	SA with PR (decrease); SA with FR (no change)
Schwartz et al. [63]	Rat	SW	SNI	AAT (decrease)

Abbreviations: SW, sucrose-containing water; SNI, spinal nerve injury; SPT, sucrose preference test; CCI, chronic constriction injury; CFA, complete Freund's adjuvant injection; SNL, spinal nerve ligation; SA, self-administration; PR, progressive ratio protocol; FR, fixed ratio protocol; PSNL, partial ligation of the sciatic nerve; AAT, avoidance-reward approach task.
